# Research on Deep Learning-Based Multi-Level Cross-Domain Foreign Object Detection in Power Transmission Lines

**DOI:** 10.3390/s25165141

**Published:** 2025-08-19

**Authors:** Qingxue Liu, Xia Wang, Yun Su, Wei Jiang, Zhe Zhang, Fuyu Shen, Lizitong Zhu

**Affiliations:** 1 School of Mechanical and Electrical Engineering, Kunming University, Kunming 650214, China; qxliu@kmu.edu.cn (Q.L.); argayun@163.com (Y.S.); 18388582739@163.com (W.J.); 13887609139@163.com (Z.Z.); 13330418727@163.com (F.S.); 2 Yunnan Key Laboratory of Intelligent Logistics Equipment and Systems, Kunming 650214, China; 3 School of Fine Art and Design, Kunming University, Kunming 650214, China; 18687815203@163.com

**Keywords:** transmission line defects, deep learning, YOLO, CNN, CO-YOLO

## Abstract

With the rapid advancement of deep learning technology, deep learning-based methods have become the mainstream approach for detecting potential safety hazards in transmission lines, playing a crucial role in power grid safety monitoring. However, existing models are often overly complex and struggle with detecting small or occluded targets, limiting their effectiveness in edge-device deployment and real-time detection scenarios enhanced the YOLOv11 model by integrating it with the ConvNeXt network, a multi-level cross-domain analysis detection model (ConvNeXt-You Only Look Once) is proposed. Additionally, Bayesian optimization was employed to fine-tune the model’s hyperparameters and accelerate convergence. Experimental results demonstrate that CO-YOLO mAP@0.5 reached 98.4%, mAP@0.5:0.95 reached 66.1%, and FPS was 303, outperforming YOLOv11 and ETLSH-YOLO, in both accuracy and efficiency. Compared with the original model, CO-YOLO model improved by 1.9% in mAP@0.5 and 2.2% in mAP@0.5:0.95.

## 1. Introduction

As the core component of modern energy transmission, the stability and safety of power systems play a crucial role in ensuring the smooth operation of society [1,2,3] reducing—or even eliminating—grid faults has become an unwavering goal pursued by smart grid technology research teams worldwide. Among these challenges, foreign object intrusion is particularly noteworthy [4,5,6]. Foreign objects on transmission lines—such as bird nests, kites, balloons, plastic waste, and branches—often adhere to the lines due to aerodynamic forces [7,8,9]. These intrusions not only compromise the insulating properties of the line surfaces but also, through friction and collision with line components, can readily trigger short circuits, line breaks, electrical fires, or even large-scale power outages, thereby posing significant safety hazards to the reliable operation of power grids [10,11]. Consequently, the timely detection and elimination of potential safety risks on transmission lines is of paramount importance.

Traditional methods for detecting foreign objects on transmission lines primarily rely on periodic manual inspections and rudimentary image processing techniques to analyze transmission line images. These approaches are not only inefficient but also fail to achieve comprehensive coverage and real-time monitoring [12]. In recent years, the rapid development of deep learning technology has led to significant advances in image recognition and object detection. Research has predominantly focused on using convolutional neural networks (CNNs) to extract features from transmission line images and identify foreign objects [13,14]. Deep learning-based object detection methods can automatically learn image features, thereby markedly improving detection accuracy. These methods generally fall into two main types: two-stage networks that utilize candidate frames [15,16,17] and one-stage networks that rely on regression [18,19,20]. Notably, the YOLO model has become a research hotspot due to its fast detection speed and its ability to generate detection results for all objects with a single forward pass, thus enabling real-time detection. However, existing YOLO models rely on multi-scale feature maps to detect objects of various sizes and tend to emphasize overall object features. As a result, the relatively large scale of the detection layers makes it difficult to capture fine details of small objects, leading to lower recognition rates for occluded or variably sized images. Moreover, their high computational complexity when processing high-resolution images hampers real-time performance on detection devices [21,22]. To address these challenges, several approaches have been proposed. For example, Tu et al. [23] introduced a dual-branch downsampling module (DBD) into the neck network of YOLOv8 and integrated a mixed enhanced attention module (MIX) within the backbone to address issues arising from variable object scales and indistinct features; however, the large number of computational parameters restricts its deployment on smaller devices. Similarly, WANG et al. [24] incorporated deformable convolution (DCN) and the parameter-free SimAM attention mechanism into the ELAN-S module of YOLOv7-Tiny to enhance the model’s capability to extract features from irregularly shaped and intra-class diverse foreign objects (e.g., garbage bags and branches). They further optimized hyperparameters using genetic algorithms (GA) and employed space-to-depth (SPD) convolutions to improve the recognition of low-resolution and small objects while accelerating model convergence. Nonetheless, the extra parameters introduced by the DCN module and the insufficient extraction of critical features by SimAM—especially when processing complex textures and multi-scale features—have limited overall performance. Additionally, Li et al. [25] augmented the YOLOv7 model by incorporating a Large Selective Kernel Network (LSKNet) structure and introducing weighted spatial attention (WSA) after the final C2f module to enhance the detection of occluded objects; yet, WSA diminished the recognition rate for irregular targets (such as garbage bags and bird nests) by overemphasizing target regions. Moreover, Yu et al. [26] introduced a channel–spatial decoupling downsampling module (CSDovn) alongside a coordinate attention (CA) mechanism and the Mish activation function to significantly reduce the parameter count and computational complexity of the YOLOv7 model, although its performance in small object detection remains unsatisfactory. Despite these efforts to integrate attention mechanisms and feature fusion modules to bolster the extraction of multi-scale object features, current methods still face limitations by not fully leveraging multi-dimensional information—such as the appearance characteristics of objects and the spatial relationships among them.

Given this, we present a novel cross-domain detection model that integrates transmission line visual features—extracted via an improved YOLOv11—with spatial appearance cues derived from ConvNeXt-B. This design enables multi-level semantic fusion, effectively enhancing the recognition of small and occluded objects in complex scenes while maintaining high real-time performance. Additionally, we incorporate Bayesian optimization to fine-tune hyperparameters automatically, which significantly accelerates model convergence and improves generalization capability. Extensive experimental validation demonstrates the effectiveness of our method in practical transmission line inspection tasks.

The key contributions of this study are summarized as follows:

1. A structural enhancement of YOLOv11, used C3k2-Dual module, NonLocalBlockND module, C2PSA-DHSA module, DySample module, PIoU and combined ConvNeXT, enabling improved detection of small, dense, and occluded targets with faster inference, tailored for real-time transmission line scenarios.
2. A novel multi-level cross-domain fusion framework is proposed that combines object-level features and spatial semantic cues using ConvNeXt-B, thereby strengthening feature representation and detection robustness.
3. Integration of Bayesian optimization for automated hyperparameter tuning, leading to improved convergence speed and overall detection performance.

Section 2 introduces the original network model along with the proposed methods and their implementation. Section 3 presents a comparative analysis between the proposed approach and several existing methods. Section 4 concludes the paper with pertinent observations and future work direction.

## 2. Models and Methods

### 2.1. Object Detection Part

**Figure 1:**
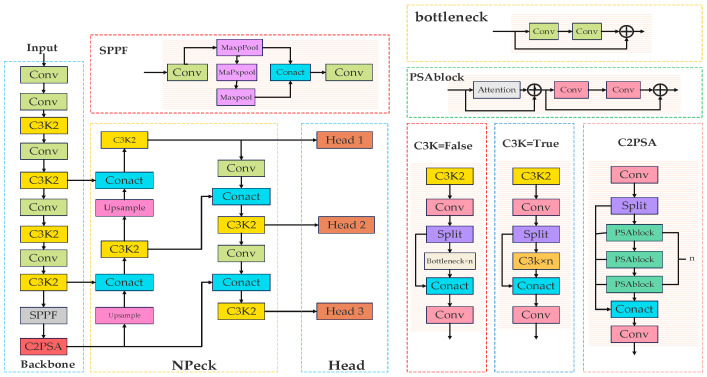
YOLOv11 network structure.

Object Detection Part consists of YOLOv11, which is mainly composed of three main parts: backbone, neck, and head, as shown in Figure 1.

The backbone is mainly used for data enhancement. The backbone is composed of multiple Conv and C3k2 modules and an SPPF module and a C2PSA module, which are mainly used to extract features from images. The neck network adopts the PAN structure and mainly consists of Upsample modules, C3k2 modules as well as Conv to achieve a multi-scale object detection effect. The head is mainly composed of multiple detection heads, which are responsible for predicting the object position and category based on the feature information refined by the neck. In this paper, C3k2 modules, C2PSA modules, Upsample modules, NonLocalBlockND modules, Loss functions are improved in five aspects concerning the characteristics of the transmission line foreign object intrusion dataset.

#### 2.1.1. C3k2-Dual Module

**Figure 2:**
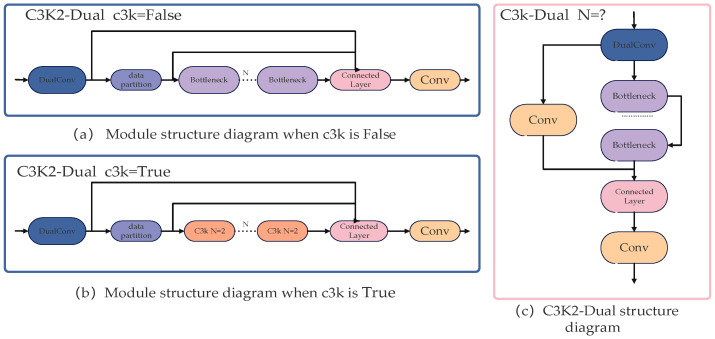
The C3 k2-Dual module structure.

The C3k2 module extracts feature information by calculating the interactions between all positions in the feature map, resulting in a quadratic increase in computational complexity. The structure is shown in Figure 2. To address this challenge, the C3k2-Dual module replaces traditional convolution (Conv) with Dual Conv, substantially enhancing computational efficiency. By incorporating grouped convolution, this design optimally arranges convolutional filters, reducing both computational cost and parameter count while improving model accuracy.

**Figure 3:**
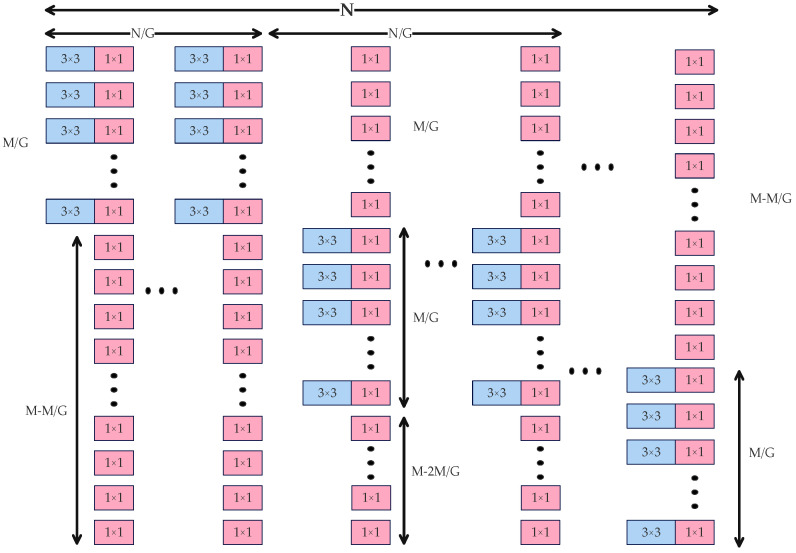
The dual-core convolution principle.

In detail, DualConv partitions N convolutional kernels into G groups (G is adjustable to balance the proportion of grouped convolutions, Process M parallel image channels simultaneously, thereby optimizing floating-point operations (FLOPs)), as shown in Figure 3. Each group processes the entire input feature map as follows: M/G input channels undergo parallel 3 × 3 and 1 × 1 convolutions to retain complete feature information, ensuring effective deep feature extraction, while the remaining (M-M/G) channels are processed exclusively by 1 × 1 convolutions to minimize the parameter burden. The resulting outputs from the 3 × 3 and 1 × 1 convolutions are summed to obtain the final feature map.

#### 2.1.2. NonLocalBlockND Module

**Figure 4:**
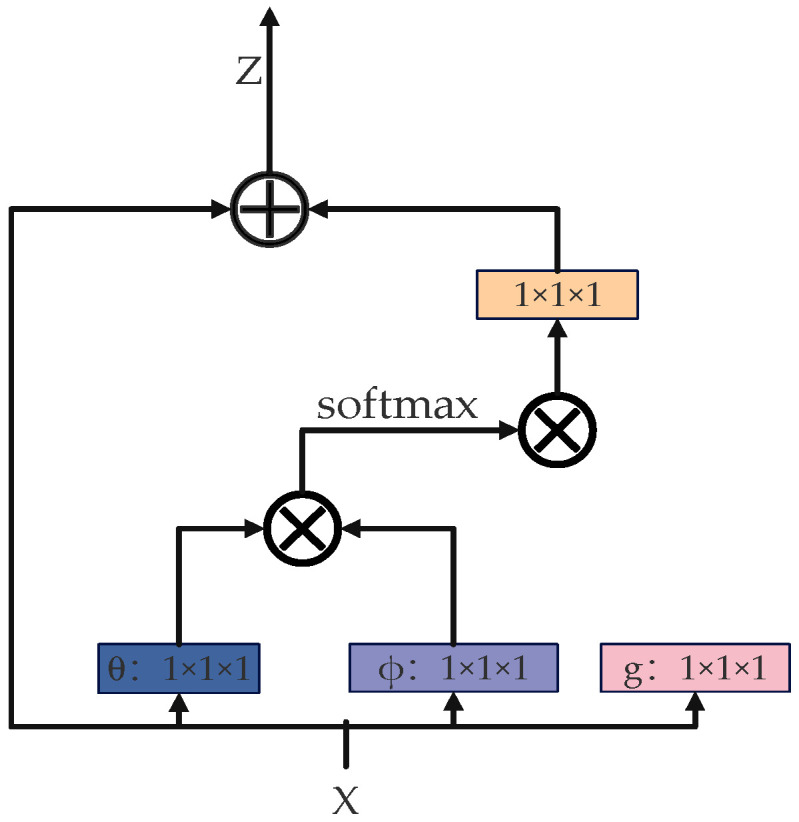
Principle of the non-local attention mechanism.

The NonLocalBlockND module improves model performance by capturing long-range dependencies across the feature map, as shown in Figure 4. Its key mechanism leverages three branches (θ, ϕ, and g) to derive downsampled feature representations, enabling similarity computation and weighted aggregation. Specifically, the θ branch generates query vectors, the ϕ branch produces key vectors for similarity estimation, and the g branch generates value vectors for weighted aggregation. The similarity between θ and ϕ produces an attention weight matrix, which, when multiplied by g, yields globally weighted feature representations. Finally, a 1 × 1 convolution restores the original channel dimensions, while a residual connection fuses the weighted and original features, effectively integrating local and global context information and significantly enhancing the model’s perceptual capabilities.

#### 2.1.3. C2PSA-DHSA Module

**Figure 5:**
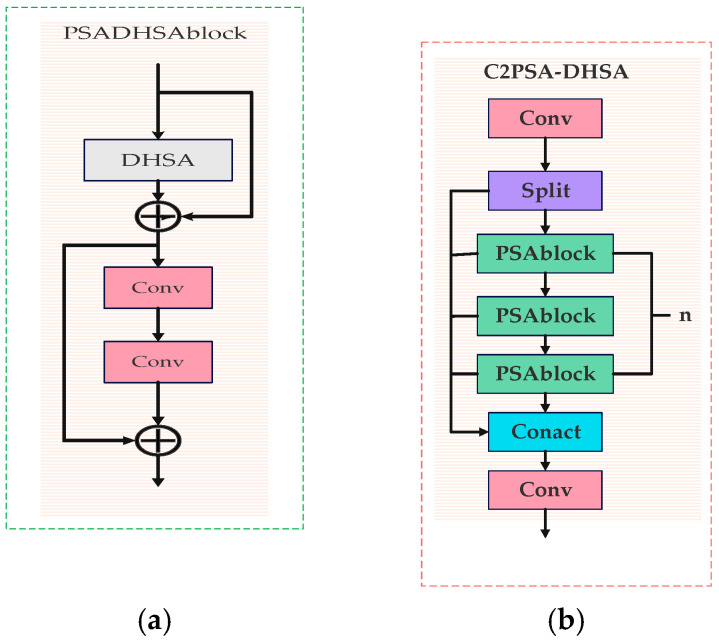
C2PSA-DHSA module principle: (**a**) PSADHSA module structural; (**b**) Structure of the C2PSA-DHSA module.

**Figure 6:**
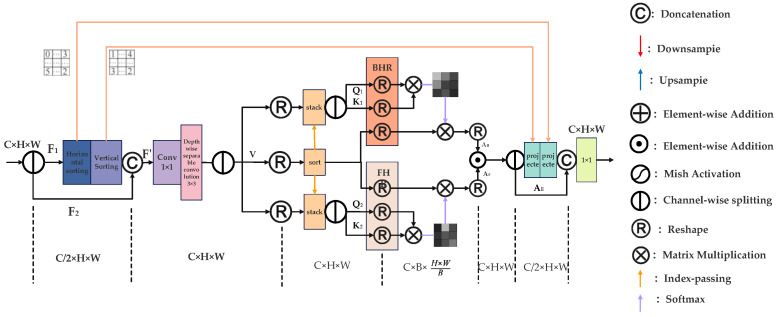
Principles of the attention mechanism of DHSA.

The conventional C2PSA module enhances the model’s attention to critical image regions by stacking multiple PSA modules. However, this design overemphasizes spatial correlations within feature maps, leading to suboptimal generalization. To address this issue, this study replaces the attention mechanism in the C2PSA module with the DHSA module, significantly improving image restoration performance, as shown in Figure 5a. The structures of the C2PSA-DHSA and DHSA modules are illustrated in Figure 5b and Figure 6.

In the proposed C2PSA-DHSA module, we aim to overcome the limitations of conventional spatial attention mechanisms that often focus too narrowly on specific regions or suffer from overfitting due to excessive parameterization.

The module begins by splitting the input feature map F into two branches (F1 and F2). The F1 branch undergoes dynamic-range sorting along both horizontal and vertical axes, generating an ordered representation that preserves spatial distribution characteristics. The sorted feature map is then concatenated with F2, which retains the original unaltered spatial information, forming a new intermediate feature map F’. This combined representation is processed through depthwise separable convolution, enhancing the module’s ability to capture long-range dependencies while reducing computational overhead. Following this, histogram-based reshaping operations—Bilateral Histogram Recalibration (BHR) and Frequency Histogram Recalibration (FHR)—are applied to dynamically balance global structures and local details, adapting to the complexity of transmission line imagery.

Finally, a self-attention mechanism fuses these enriched features, enabling the network to better model semantic relevance and spatial dependencies. This refined attention fusion significantly improves the quality of feature extraction, particularly for irregular and occluded foreign objects.

#### 2.1.4. DySample Module

**Figure 7:**
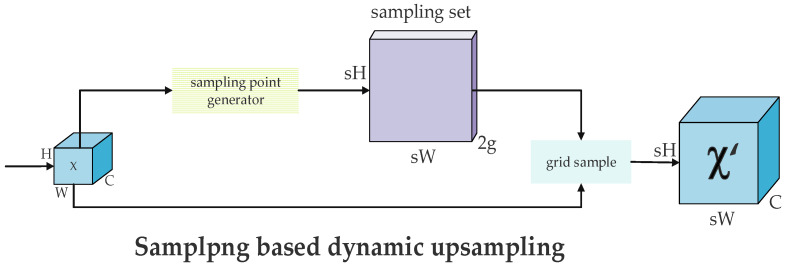
The DySample module structure.

**Figure 8:**
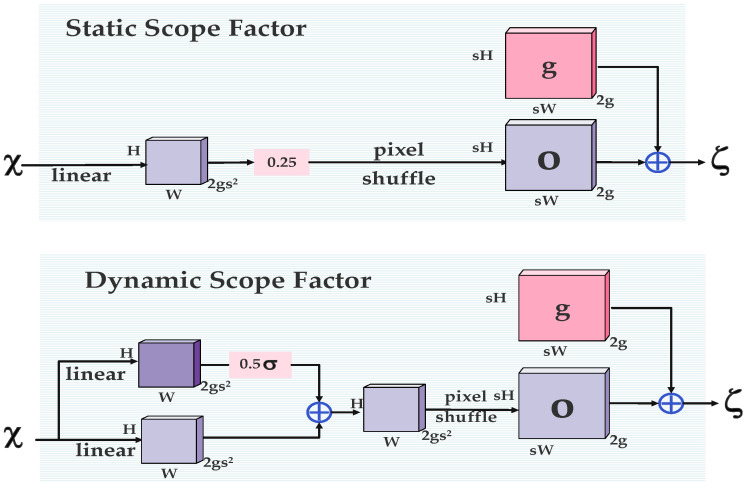
Two different range factors.

In the YOLOv11 framework, the feature upsampler applies a fixed interpolation rule to convert low-resolution feature maps into high-resolution counterparts, as delineated in Equation (1). However, this method, which depends on adjacent pixel spacing, achieves mere spatial enlargement of the feature map without adequately preserving its detailed features or semantic content. Consequently, this limitation hampers the model’s performance in dense prediction tasks, such as foreign object detection in transmission lines. To overcome this shortfall, we introduce an advanced upsampling module, termed DySample. Departing from conventional fixed-rule interpolation, DySample employs a dynamic point-sampling strategy. Its operational mechanism is elucidated in Equation (2), with its architecture depicted in Figure 7. Within DySample, the F1 branch dynamically learns the coordinates of sampling points across the feature map. To regulate these points, a range factor—illustrated in Figure 8—is incorporated to limit the offset range of the sampling positions, yielding a refined sampling set, *S*. Subsequently, the feature map *X* is resampled using the grid_sample function based on the coordinates defined in *S*.

(1) $$X_{\mathrm{high}}=\mathrm{Interp}(X_{\mathrm{low}}),$$

(2) $$X_{\mathrm{high}}=\mathrm{GridSample}(X_{\mathrm{low}},S),$$

#### 2.1.5. Loss Function

Although YOLOv11 deviates from traditional approaches by employing the SIoU (Scale-Aware Intersection over Union) loss function instead of CIoU to improve the detection of small objects, this shift inadvertently introduces errors in computing overlapping regions between objects. As a result, the model’s localization precision and overall detection performance are markedly reduced. To mitigate this limitation, we propose replacing SIoU with the *PIoU* (Powerful Intersection over Union) loss function, the formulation of which is presented in Equation (3), $B_{p}$ represents the Predicted Box, and $B_{g}$ represents the Ground Truth Box. $\left|B_{p}\cap B_{g}\right|$ is the area of the intersection between the predicted box and the ground truth box, while $\left|B_{p}\cup B_{g}\right|$ is the area of their union. The *PIoU* loss function quantifies the overlap between predicted and ground-truth bounding boxes by calculating their IoU value. while additionally accounting for angular discrepancies between them. This enhancement refines the bounding box regression process, enabling superior adaptation to objects with high aspect ratios or those embedded in complex backgrounds.

(3) $$L_{PIoU}=1-\frac{\left|B_{p}\cap B_{g}\right|}{\left|B_{p}\cup B_{g}\right|},$$

#### 2.1.6. Improved Network Model

**Figure 9:**
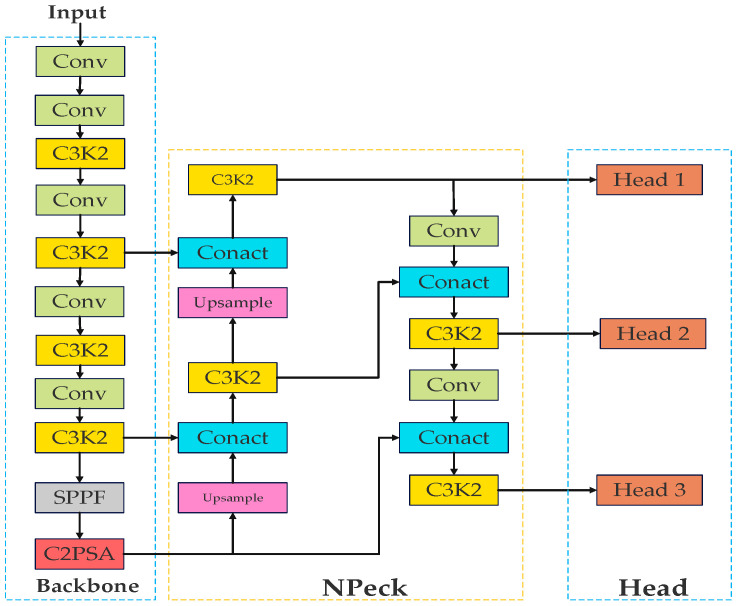
The refined YOLOv11 structure.

In this paper, we mainly enhance the CO-YOLO backbone network by integrating three key modifications: the C3k2-Dual Module, the C2PSA-DHSA Module, and the NonLocalBlockND Module (added before the SPPF module) to refine feature extraction. Additionally, the DySample module is employed to enhance the upsampling capability of the neck, while Powerful-IoU Loss is incorporated into the detection head to improve detection performance. These improvements significantly enhance CO-YOLO’s ability to detect foreign objects in power transmission lines. The overall improved network structure is illustrated in Figure 9.

### 2.2. Relationship Detection

#### ConvNeXT-B

**Figure 10:**
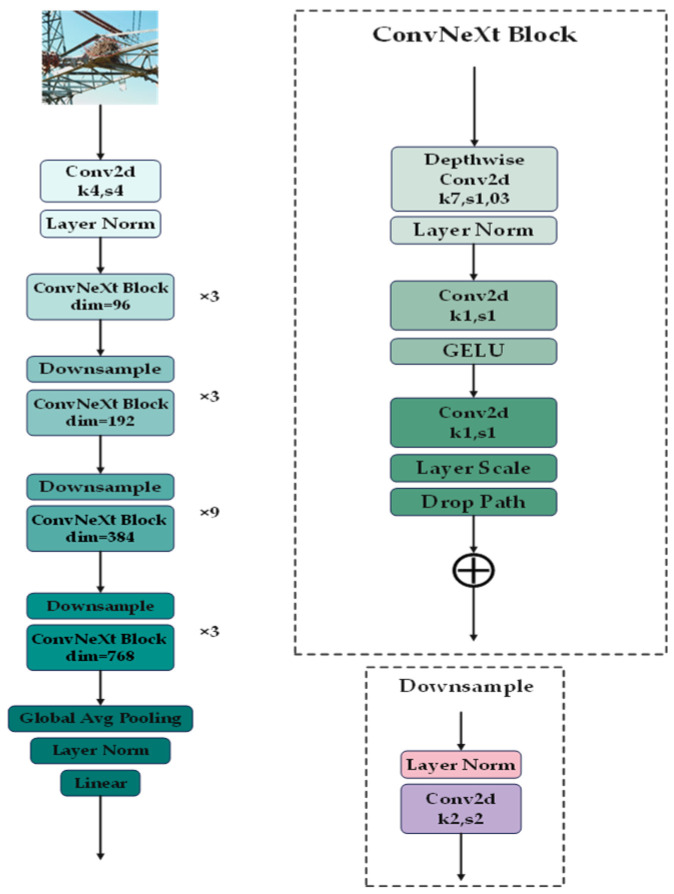
ConvNeXt network structure.

As shown in Figure 10, ConvNeXt extracts features by stacking multiple ConvNeXt blocks. In this study, we employ ConvNeXt to capture the feature relationships of foreign objects, enabling the recognition model to acquire richer appearance features and their spatial dependencies. This approach significantly enhances the network’s feature extraction capability, allowing it to better understand both the visual characteristics and spatial relationships of the detected objects.

### 2.3. CO-YOLO

**Figure 11:**
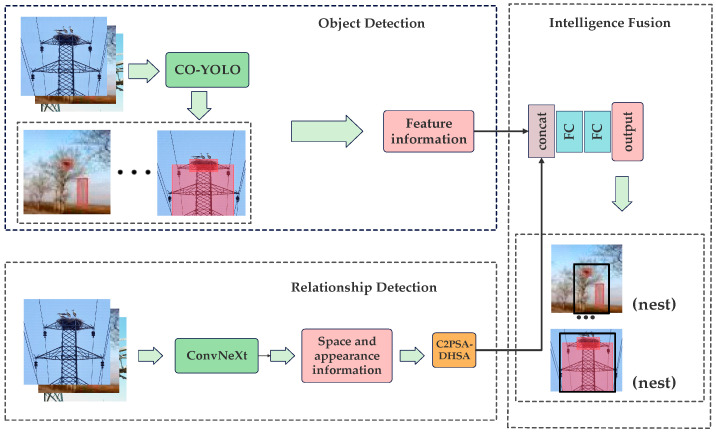
The CO-YOLO network structure.

As shown in Figure 11, ConvNeXt extracts features by stacking multiple ConvNeXt blocks. In this study, we utilize ConvNeXt to capture the feature relationships of foreign objects, enabling the recognition model to acquire richer appearance features and their spatial relationships, thereby enhancing the network’s feature extraction capability.

## 3. Experiments and Analyses

### 3.1. Experimental Settings

Table 1 summarizes the primary environment configurations used in our experiment, including the operating system, CPU, GPU, and the versions of Python, CUDA, and PyTorch. In order to optimize the hyperparameters of the model, we adopted Bayesian optimization and constructed the posterior distribution of the objective function by using Bayes theorem. By collecting functions (such as expected improvement EI, probability improvement PI, and upper confidence bound UCB), we selected the next combination of hyperparameters for evaluation based on the posterior distribution, so as to approximate the optimal value with fewer evaluations.

The final optimized settings were: optimizer = SGD, batch size = 16, initial learning rate (lr0) = 0.2, final learning rate (lrf) = 0.01, momentum = 0.937, weight decay = 0.0005, warmup epochs = 20, warmup momentum = 0.8, and total epochs = 350. All remaining hyperparameters were set to the default values provided by the official YOLOv11 implementation, which served as the baseline model.

### 3.2. DataSets

**Figure 12:**
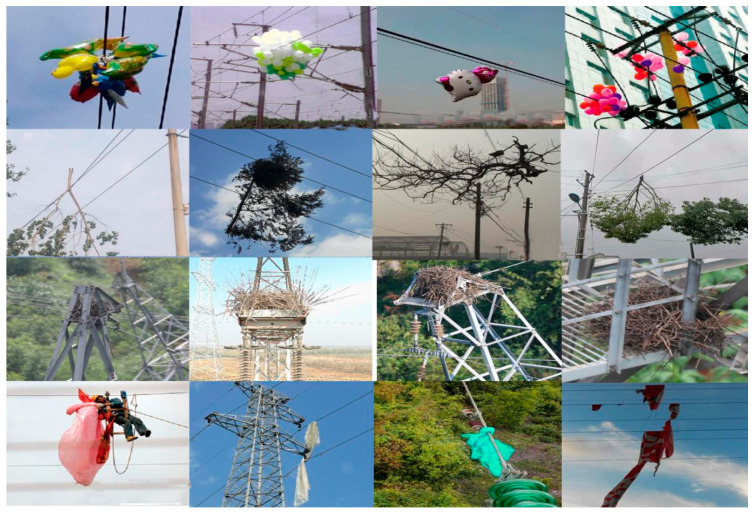
Part of the dataset.

Due to the lack of publicly available datasets on foreign object intrusions in cross-domain transmission lines, we compiled a custom dataset of 979 images by integrating data from existing public datasets, media resources, and manually collected samples. This dataset includes four categories of foreign objects: bird nests, balloons, branches, and plastic debris. Given the diverse and complex nature of these intrusions, a larger volume of image data is essential for effective feature learning by the model. To address this, we applied a range of data augmentation techniques, such as random cropping, flipping, and rotation, to expand the dataset. Furthermore, we simulated images captured under adverse conditions by applying random grayscale transformations and adjusting hue, saturation, exposure, and brightness. As a result of this augmentation process, the dataset size increased to 2739 images. A detailed overview of the dataset is provided in Table 2 and Figure 12.

The dataset covers a variety of typical transmission line scenarios and fault types. During the data collection process, strict adherence to industry standards and practical application requirements is maintained to ensure the diversity and representativeness of the samples.

Data preprocessing includes image denoising, format unification, and removal of abnormal samples. The labeling process is conducted by a team of engineers with professional backgrounds, and a multi-round review mechanism is employed to ensure the accuracy of the labeling. The labeling content mainly includes the position boxes and category labels of the target objects.

For data quantification, we divide the dataset according to the following criteria:

- Training set: 70% of the total samples, used for model parameter training;
- Validation set: 15%, used for hyperparameter tuning and model selection;
- Test set: 15%, used for final performance evaluation.

### 3.3. Evaluation Metrics

In our experiments, we used several metrics to evaluate the model’s performance, including accuracy, precision, recall, average precision (AP), mean average precision (mAP), confusion matrix, parameter count, GFLOPs, and frames per second (FPS). Among these, mAP serves as a key performance indicator, especially for object detection tasks.

The mean Average Precision (mAP) is often divided into two metrics: mAP@0.5 and mAP@0.5:0.95. Here, mAP@0.5 indicates the mean Average Precision (AP) across all categories when the Intersection over Union (IoU) threshold is fixed at 0.5. In contrast, mAP@0.5:0.95 represents the average AP calculated over a series of IoU thresholds ranging from 0.5 to 0.95, with an incremental step of 0.05. Given K categories where K > 1, the mAP can be formulated as Equation (4):

(4) $$mAP=\frac{1}{K}\sum_{k=1}^{K}AP_{k},$$

where $AP_{k}$ is the Average Precision for category K. The AP itself is calculated by integrating the area under the Precision-Recall curve, which reflects the balance between precision and recall across different decision thresholds.

In addition to mAP, the Confusion Matrix provides a detailed breakdown of the model’s predictions, helping to visualize true positives (TP), false positives (FP), true negatives (TN), and false negatives (FN). From this, important evaluation metrics like accuracy, precision, and recall are derived:

- *Accuracy* measures the proportion of total predictions that are correct, defined as Equation (5) as follows:

(5) $$Accuracy=\frac{TP+TN}{TP+TN+FP+FN},$$

- *Precision* reflects the proportion of predicted positive samples that are indeed positive shown as Equation (6) as follows:

(6) $$Precision(P)=\frac{TP}{TP+FP},$$

- *Recall* (also called Sensitivity) captures the proportion of actual positive samples correctly identified, shown as Equation (7) as follows:

(7) $$Recall(R)=\frac{TP}{TP+FN}$$

Furthermore, Parameter Count (parameters) and GFLOPs (giga floating point operations per second) evaluate the computational complexity of the model. A lower parameter count and fewer FLOPs generally indicate a more lightweight and efficient model. FPS (frames per second) measures the real-time inference speed of the model, an essential metric for deployment in time-sensitive applications.

### 3.4. Benchmark Comparison Experiment

**Figure 13:**
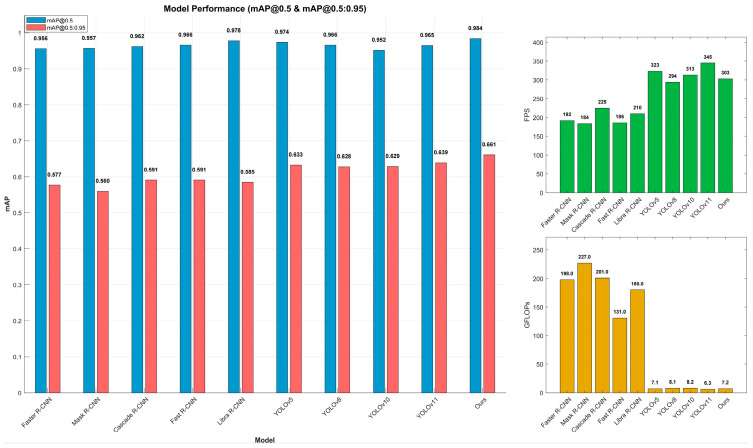
Histogram of basic model experimental values.

Table 3 and Figure 13 present a comparison of key detection models, highlighting the superior parameter efficiency and FPS of single-stage detection models over their two-stage counterparts. The proposed CO-YOLO model demonstrates significant advantages over other YOLO series models (YOLOv5, YOLOv8, YOLOv10, and YOLOv11). Compared to YOLOv5, our model improves mAP@0.5 by 1.0% and mAP@0.5:0.95 by 2.8%, with a 12.7% increase in parameter count and a 1.4% rise in GFLOPs, while FPS only decreases by 6.0%. Similarly, compared to YOLOv11, CO-YOLO achieves a 1.9% boost in mAP@0.5 and a 2.2% increase in mAP@0.5:0.95, with a 9.2% rise in parameters, a 14.3% increase in GFLOPs, and a 12.1% drop in FPS. These results demonstrate that CO-YOLO maintains high real-time performance while significantly enhancing detection accuracy and robustness, particularly for challenging objects like bird nests and branches. This makes it a more efficient and reliable solution for foreign object intrusion detection in power transmission lines.

As shown in Table 4, we compared the performance of four different attention mechanisms to evaluate their impact on the model’s effectiveness. Notably, the NonLocalBlockND attention mechanism achieved the best results in both mAP@0.5:0.95 and FPS. Compared to the most parameter-efficient attention mechanism, NonLocalBlockND increased the parameter count by only 3.45%, while improving mAP@0.5:0.95 by 1.72%, mAP@0.5 by 0.61%, and FPS by 2.8%. These results demonstrate that NonLocalBlockND effectively integrates local and global contextual information, significantly enhancing the model’s perception capabilities. Considering both recall and precision, it is evident that the proposed algorithm outperforms other types of methods, thereby validating its effectiveness.

To comprehensively evaluate the impact of various improvements on model performance, we compared the effects of the C2PSA module, C3K2 module, and different loss functions, as shown in Table 5, Table 6 and Table 7.

By comparing different C2PSA variants, we found that the C2PSA-DHSA module outperforms the C2PSA-DAT module by enhancing target extraction capabilities while reducing parameter count. Specifically, the C2PSA-DHSA module decreases parameter count by 4.29%, while improving mAP@0.5 by 0.31% and mAP@0.5:0.95 by 0.63%. This demonstrates that the C2PSA-DHSA module significantly improves feature extraction efficiency while reducing computational cost. Further analysis of Table 6 shows that the C3K2-Dual module achieves the highest mAP@0.5 and mAP@0.5:0.95 among all variants, with improvements of 1.04% and 1.74%, respectively. This highlights the C3K2-Dual module’s outstanding performance in object detection, effectively enhancing model accuracy and robustness. Moreover, as shown in Table 7, the PIoU loss function achieves the best results in parameter efficiency, recall, and FPS. Notably, compared to the EIoU loss, PIoU reduces the parameter count by 5.28% while maintaining the same recall rate. This result indicates that the PIoU loss function reduces model complexity without compromising sensitivity, improving both efficiency and accuracy—aligning well with the principles of efficient module selection.

### 3.5. Subsection

**Figure 14:**
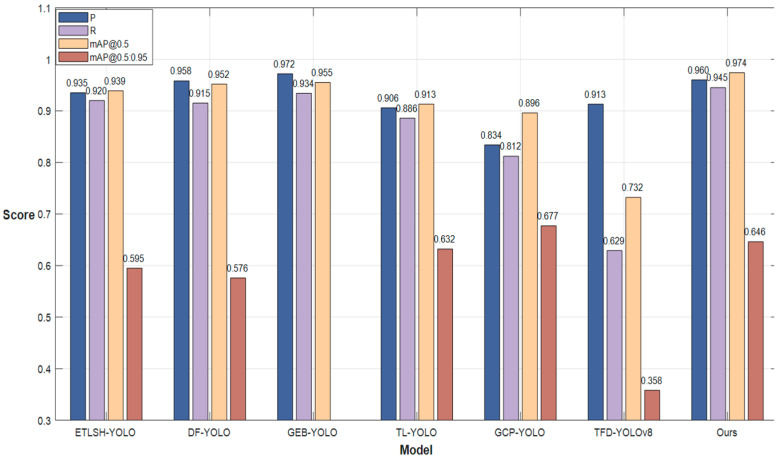
Other high-level modules compare data histograms.

As shown in Table 8 and Figure 14, the proposed CO-YOLO model was compared against several state-of-the-art algorithms for foreign object intrusion detection in power transmission lines. The experimental results demonstrate that CO-YOLO outperforms all other models in the key metrics of mAP@0.5 and mAP@0.5:0.95. Specifically, compared to ETLSH-YOLO, CO-YOLO improves mAP@0.5 by 3.7% and mAP@0.5:0.95 by 8.6%. Against DF-YOLO, the improvements are 2.3% and 12.2%, respectively. Compared to GEB-YOLO, CO-YOLO achieves a 2.0% gain in mAP@0.5 and a 12.2% increase in mAP@0.5:0.95. The model also surpasses TL-YOLO by 6.7% in mAP@0.5 and 2.2% in mAP@0.5:0.95. While CO-YOLO achieves an 8.7% higher mAP@0.5 compared to GCP-YOLO, its mAP@0.5:0.95 is 4.7% lower. Finally, compared to TFD-YOLOv8, CO-YOLO shows remarkable gains of 33.0% in mAP@0.5 and 80.4% in mAP@0.5:0.95. These results clearly highlight the superior detection accuracy and robustness of CO-YOLO, making it more effective at identifying foreign objects in power transmission lines and providing a more reliable safeguard for the safe operation of transmission infrastructure.

### 3.6. Ablation Study

To accurately evaluate the impact of different modules on model performance, validate the effectiveness of the design, and explore the relationships between the modules, we conducted a series of ablation experiments based on the YOLOv11 model. To ensure the scientific rigor and reliability of the results, all experimental settings were kept consistent, with the results summarized in Table 9.

Compared to the baseline YOLOv11 model, the integration of the C2PSA-DHSA module increased mAP@0.5 by 0.6% and decreased mAP@0.5:0.95 by 0.3%, while increasing the parameter count by 2.09% (from 2,582,932 to 2,637,080) and GFLOPs by 1.59% (from 6.3 to 6.4). The addition of the DySample module further boosted mAP@0.5 to 0.974 and mAP@0.5:0.95 to 0.638, while reducing the parameter count by 1.58% and lowering GFLOPs by 1.56%. When used independently, the C3K2-Dual module achieved the best mAP@0.5 (0.979) and mAP@0.5:0.95 (0.642), improving performance by 1.4% and 0.5%, respectively, compared to models without this module. The combination of the NonLocalBlockND, C2PSA-DHSA, and C3K2-Dual modules pushed mAP@0.5 to 0.982 and mAP@0.5:0.95 to 0.654, representing gains of 0.3% and 1.2%, respectively, over models without NonLocalBlockND. Finally, the introduction of a specific loss function resulted in the highest performance, with mAP@0.5 reaching 0.984 and mAP@0.5:0.95 reaching 0.641—improvements of 0.2% and 0.3% compared to models without this loss function. These results clearly demonstrate that the combination of these modules and the optimized loss function significantly enhances model performance while maintaining relatively low computational complexity, providing an efficient and reliable solution for foreign object intrusion detection in power transmission lines.

Through a comprehensive series of ablation experiments, we found that the combination of these modules and the optimized loss function significantly improves the performance of the YOLOv11 model while maintaining low computational complexity. Ultimately, the proposed CO-YOLO model achieved the best performance across all experiments, offering an efficient and accurate solution for foreign object detection in power transmission lines.

### 3.7. Attention Visualization Comparison Experiment

**Figure 15:**
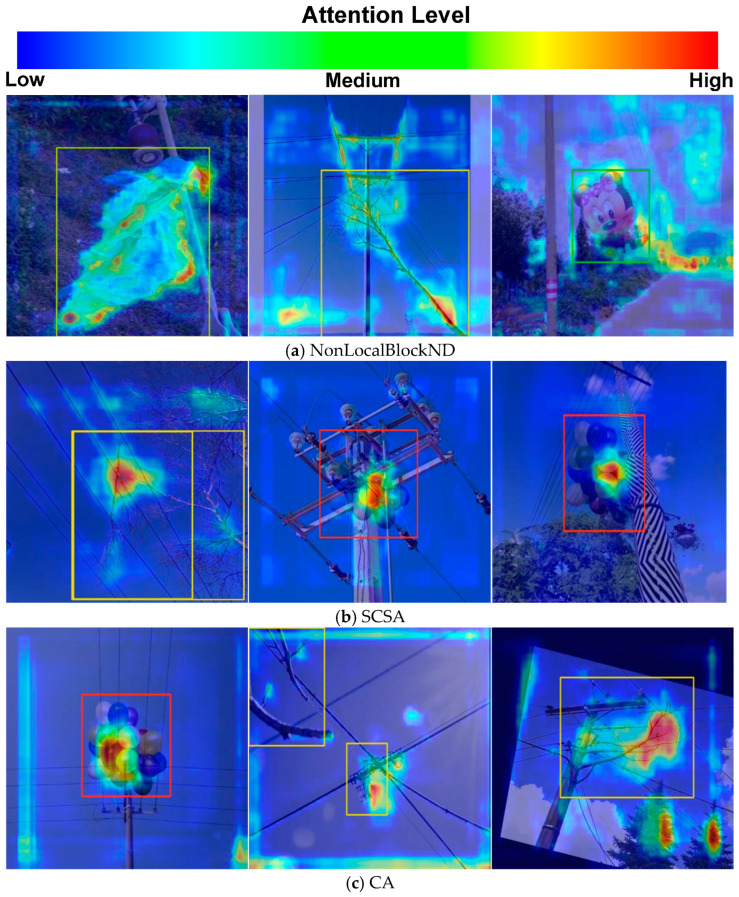
Visual comparison of the attention mechanism.

In the field of deep learning, attention mechanisms have been widely applied to various tasks to enhance a model’s ability to focus on key information, thereby significantly improving performance. In this study, we conducted a systematic visual comparison of different attention mechanisms by analyzing their focus regions and weight distributions, highlighting the strengths and weaknesses of each approach. As shown in Figure 15, the NonLocalBlockND attention mechanism evenly distributes attention across the target object, allowing the model to capture both local and global contextual information effectively. In contrast, the SCSA attention mechanism suffers from over-concentration, focusing excessively on a small portion of the target object, which limits the model’s ability to capture comprehensive features. This weakness is particularly evident when detecting objects like balloons, where both local and global information are crucial for accurate recognition. Similarly, the CA mechanism also exhibits over-concentration issues, often directing attention toward irrelevant objects, thereby weakening the model’s feature extraction capabilities. The results clearly demonstrate that NonLocalBlockND effectively distributes attention across transmission lines and foreign objects, integrating global information to help the model better understand the relationship between the target and its background—a key requirement for foreign object detection in power transmission lines.

### 3.8. Generalized Experiments

To verify the model’s performance in other fields or with different datasets and actual operation, we conducted validation experiments using FOTL (China Insulator Dataset, including insulator data from railway, communication, and power sectors) and CPLID (Southeast Asian Power Industry Dataset, including bird nests, branches, flying objects, and regional flora and fauna characteristics). The experimental results are shown in Table 10.

Table 10 states that the provided data shows the proposed improved model in this study demonstrates significant advantages over the FOTL and CPLID datasets on self-built datasets. Compared to the FOTL dataset, the Ours model improves accuracy (P) by 4.3%, recall rate (R) by 2.0%, mAP@0.5 by 4.1%, and mAP@0.5:0.95 by 6.4%. In comparison with the CPLID dataset, P improves by 6.9%, R by 9.8%, mAP@0.5 by 6.0%, and mAP@0.5:0.95 by 24.0%. These results indicate that the self-built dataset has advantages in data diversity and annotation quality, better supporting model training and optimization. Additionally, the model achieves an mAP@0.5 of 0.945 and 0.928 on the FOTL and CPLID datasets, respectively, demonstrating good adaptability; mAP@0.5:0.95 reaches 0.621 and 0.533, both superior to YOLOv8 and YOLOv10 models.

## 4. Conclusions

In this study, we proposed a multi-level cross-domain foreign object detection model tailored for power transmission lines. By integrating the YOLOv11 backbone with ConvNeXt-B and employing Bayesian optimization for hyperparameter tuning, the model effectively enhances detection accuracy and real-time performance, particularly for small and occluded targets, outperforming to of the classical models as in as follows

1. Compared to YOLOv11, CO-YOLO improves mAP@0.5 by 1.9% mAP@0.5:0.95 by 2.2%, with only a 12.1% reduction in FPS.
2. Compared to ETLSH-YOLO, it achieves improvements of 3.7% and 8.6% in mAP metrics.
3. When tested against TFD-YOLOv8, mAP@0.5 and mAP@0.5:0.95 increased by 33.0% and 80.4%, respectively.

CO-YOLO achieves 98.4% mAP@0.5, 66.1% mAP@0.5:0.95, and 303 FPS, outperforming all compared models.

The model also demonstrates excellent generalization on public datasets (FOTL, CPLID), showing gains of up to 6.4% mAP@0.5:0.95 compared to baseline models. These results highlight CO-YOLO’s high robustness, precision, and applicability in complex outdoor environments.

However, certain limitations persist. The model’s detection performance may still degrade under extreme environmental conditions (e.g., fog and snow) or for rare object types not present in the training set. To address this, we constructed a diverse dataset with simulated weather and resolution variations to enhance robustness.

Future research may also focus on: Lightweight models and acceleration, multimodal perception, and task-level generalization.

## Figures and Tables

**Table 1:** Environment configuration.

Item	Configuration
Operation System	Win11
CPU	12th Gen Intel Core^TM^ i9-12900H
GPU	NVIDIA GeForce RTX 3060 Laptop GPU (6 GB)
Python	3.8
PyTorch	2.1.2
Cuda	10.0

**Table 2:** Detailed partitioning of datasets.

	Type	Nest	Branch	Balloon	Plastic	All
Number						
Origin		315	132	352	180	979
Enhanced		576	498	679	328	2081

**Table 3:** Basic model comparison.

Model	Type	GFLOPs	Pramas	P (Precision)	R (Recall)	mAP@0.5	mAP@0.5:0.95	FPS
Faster R-CNN	Two-stage	198	4287; 5000	0.576	0.601	0.956	0.577	192
Mask R-CNN	Two-stage	227	4439; 6000	0.535	0.598	0.957	0.56	184
Cascade R-CNN	Two-stage	201	6916; 1000	0.567	0.622	0.962	0.591	225
Fast R-CNN	Two-stage	131	4070; 0000	0.596	0.618	0.966	0.591	186
Libra R-CNN	Two-stage	180	4162; 7000	0.567	0.632	0.978	0.585	210
YOLOv5	One-stage	7.1	250; 3724	**0.962**	**0.958**	0.974	0.633	323
YOLOv8	One-stage	8.1	300; 6428	0.961	0.918	0.966	0.628	294
YOLOv10	One-stage	8.2	269; 5976	0.917	0.93	0.952	0.629	313
YOLOv11	One-stage	6.3	258; 2932	0.958	0.946	0.965	0.639	**345**
Ours	One-stage	7.2	282; 1696	0.96	0.945	**0.984**	**0.661**	303

The bold part is the best value; GFLOPs = FLOPs/10^9^.

**Table 4:** Contrasts of different attention mechanisms.

Model	Pramas	P	R	mAP@0.5	mAP@0.5:0.95	FPS
CBAM	2,648,822	**0.967**	0.929	0.974	0.634	286
ECA	**2,624,535**	0.961	**0.967**	0.978	0.638	286
SCSA	2,730,908	0.959	0.962	**0.986**	0.638	278
Ours	2,715,156	0.962	0.963	0.984	**0.649**	**294**

The bold part is the best value.

**Table 5:** Comparison of different C2PSA modules.

Model	Pramas	P	R	mAP@0.5	mAP@0.5:0.95
C2PSA-ACmix	2,737,044	0.943	0.947	**0.974**	0.625
C2PSA-DAT	2,755,228	**0.971**	0.952	0.968	0.633
C2PSA-EMA	2,677,564	0.946	0.944	0.965	0.633
C2PSA-FL	2,743,900	0.881	0.856	0.921	0.545
Ours	**2,637,080**	0.955	**0.956**	0.971	**0.637**

The bold part is the best value.

**Table 6:** Comparison of different C3K2 modules.

Model	Pramas	P	R	mAP@0.5	mAP@0.5:0.95	FPS
C3K2-MLLAB	2,729,596	0.937	0.96	0.972	0.639	203
C3K2-RFA	2,953,804	0.952	**0.964**	0.969	0.643	185
C3K2-SC	**2,775,852**	**0.961**	0.95	**0.983**	0.634	250
Ours	2,877,420	0.934	0.95	0.975	**0.644**	**256**

The bold part is the best value.

**Table 7:** Comparison of the different loss functions.

Model	Pramas	P	R	mAP@0.5	mAP@0.5:0.95	FPS
EIoU	2,726,812	0.954	**0.956**	0.973	0.624	**313**
WIoU	2,726,812	0.961	0.95	**0.976**	0.635	294
DIoU	2,726,812	0.951	0.954	0.972	**0.65**	294
SIoU	2,726,812	0.954	**0.956**	0.973	0.624	303

The bold part is the best value.

**Table 8:** Comparison of the other advanced modules.

Model	P	R	mAP@0.5	mAP@0.5:0.95
ETLSH-YOLO [27]	0.935	0.920	0.939	0.595
DF-YOLO [25]	0.958	0.915	0.952	0.576
GEB-YOLO [28]	0.972	0.934	0.955	—
TL-YOLO [29]	0.906	0.886	0.913	0.632
GCP-YOLO [30]	0.834	0.812	0.896	**0.677**
TFD-YOLOv8 [31]	0.913	0.629	0.732	0.358
Ours	**0.960**	**0.945**	**0.974**	0.646

The bold part is the best value.

**Table 9:** Experimental data comparison of ablation.

Model	C2PSA-DHSA	C3k2-Dual	NonLocalBlockND	DySample	Loss	GFLOPs	Pramas	P	R	mAP@0.5	mAP@0.5:0.95	FPS
YOLOv11						6.3	**2,582,932**	**0.958**	0.946	0.965	0.639	**345**
YOLOv11	√					6.4	2,637,080	0.955	0.956	0.971	0.637	294
YOLOv11				√		6.3	2,595,284	**0.958**	0.956	0.974	0.638	270
YOLOv11	√	√				7.5	2,931,568	0.97	**0.961**	0.979	0.642	294
YOLOv11	√		√			6.5	2,769,304	0.97	0.944	0.982	0.654	270
YOLOv11	√			√		6.4	2,649,432	0.96	0.917	0.965	0.633	270
YOLOv11	√	√	√			7.6	3,063,792	0.961	0.941	0.973	**0.646**	263
YOLOv11	√	√		√		7.5	2,943,920	0.964	0.907	0.97	0.634	263
YOLOv11		√	√	√		7.6	3,021,996	0.962	0.926	0.972	0.645	286
YOLOv11	√		√	√		6.5	2,781,656	0.965	0.941	0.978	0.643	263
YOLOv11	√	√	√	√		7.6	3,076,144	0.952	0.946	0.969	0.632	263
Ours	√	√	√	√	√	7.2	2,821,696	0.96	0.945	**0.984**	0.641	303

The bold part is the best value.

**Table 10:** Comparison experiment of different datasets.

Date sets	P	R	mAP@0.5	mAP@0.5:0.95
FOTL	0.920	0.926	0.945	0.621
CPLID	0.898	0.861	0.928	0.533
Ours	0.960	0.945	0.984	0.661

## Data Availability

The data presented in this study are not publicly available due to privacy and confidentiality restrictions.
